# Cost-utility analysis of the wearable cardioverter defibrillator in high-risk post-myocardial infarction patients in the Spanish healthcare system

**DOI:** 10.3389/fcvm.2026.1832880

**Published:** 2026-05-29

**Authors:** José González Costello, Víctor Exposito Garcia, Eoin Moloney, Vasileios Kontogiannis, Mehdi Javanbakht, Farai Goromonzi, Brigitte Both, Raúl Moreno

**Affiliations:** 1 Cardiomyopathies, Advanced Heart Failure and Heart Transplant Unit, Department of Cardiology, Hospital Universitari de Bellvitge, BIOHEART Cardiovascular Diseases Research Group, IDIBELL, L’Hospitalet de Llobregat, Barcelona, Spain; 2 Department of Clinical Sciences, School of Medicine, Universitat de Barcelona, Barcelona, Spain; 3 CIBER de Enfermedades Cardiovasculares (CIBERCV), Instituto de Salud Carlos III, Madrid, Spain; 4 Arrhythmia Service, Cardiology Department, University Hospital Marques de Valdecilla, Santander, Spain; 5 Optimax Access Ltd., Southampton University Science Park, Southampton, United Kingdom; 6 ZOLL Medical Deutschland GmbH, Cologne, Germany; 7 Cardiology Department, University Hospital La Paz, Madrid, Spain; 8 Instituto de Investigación Sanitaria Hospital Universitario La Paz (IdiPAZ), Madrid, Spain; 9 Universidad Autónoma de Madrid, Madrid, Spain

**Keywords:** cost-utility analysis, myocardial infarction, Spanish healthcare system, sudden cardiac death, wearable cardioverter defibrillator

## Abstract

**Introduction:**

Patients with reduced left ventricular ejection fraction after myocardial infarction (MI) remain at increased risk of sudden cardiac death during the early post-MI period, before implantable cardioverter defibrillator eligibility is confirmed. The wearable cardioverter defibrillator (WCD) offers temporary protection during this vulnerable interval within post-MI care. We evaluated the cost-utility of WCD therapy plus guideline-directed medical therapy (GDMT) vs. GDMT alone from the Spanish healthcare system perspective.

**Methods:**

A cost-utility analysis used a Markov state-transition model with monthly cycles and a lifetime horizon for a hypothetical cohort of 1,000 post-MI patients with left ventricular ejection fraction 35% or less at discharge. Early mortality inputs were based on the as-treated VEST analysis; longer-term risks and utilities came from published sources. Direct medical costs used Spanish sources (2025 euros). Deterministic and probabilistic sensitivity analyses were conducted, including a scenario analysis using per-protocol effectiveness estimates from the VEST trial.

**Results:**

WCD plus GDMT was associated with projected gains of 0.53 life years and 0.43 quality-adjusted life years (QALYS) vs. GDMT alone (12.83 vs. 12.30 life years; 9.59 vs. 9.16 QALYs). Total lifetime costs were €113,290 vs. €102,032, giving incremental costs of €11,259 and an incremental cost-effectiveness ratio of €26,145 per QALY gained, below the €30,000 threshold. The probability of cost-effectiveness was 78.3% for patients during the early post-MI period.

**Conclusions:**

In Spain, temporary WCD use alongside GDMT after MI shows potential to improve health outcomes at an incremental cost that falls within commonly cited willingness-to-pay thresholds. These findings support adoption of WCD therapy for patients at transiently high risk of sudden cardiac death, while highlighting the importance of appropriate patient selection and adherence in clinical practice.

## 1 Introduction

Sudden cardiac death (SCD) remains a major cause of cardiovascular mortality and is most commonly the result of malignant ventricular arrhythmias in patients with underlying structural heart disease (1, 2). In Europe, SCD accounts for a substantial proportion of cardiovascular deaths, and although reported incidence in Spain may be lower than in some Northern European countries, the absolute burden remains clinically important because of the high prevalence of ischaemic heart disease and heart failure (2–5). Outcomes following out-of-hospital cardiac arrest remain poor across Europe, underscoring the importance of preventive strategies aimed at identifying and protecting high-risk patients before a cardiac arrest occurs (6–8).

Reduced left ventricular ejection fraction (LVEF) is a well-established predictor of SCD, particularly following myocardial infarction (MI) (9, 10). Patients with impaired LVEF after MI face an increased risk of SCD, with the highest vulnerability occurring during the early post-infarction period (11). Implantable cardioverter defibrillators (ICDs) have been shown to reduce arrhythmic death and improve survival in selected high-risk patients (12). However, current European guidelines recommend that primary prevention ICD implantation be considered only after an appropriate waiting period following MI, once guideline-directed medical therapy (GDMT) has been optimised and ventricular function has been reassessed (8). During this initial post-MI phase, patients with severely reduced LVEF may therefore remain exposed to residual arrhythmic risk while pharmacological treatment is being progressively up-titrated (13, 14). This early post-MI period is characterised by dynamic ventricular remodelling and clinical recovery, meaning that risk stratification at hospital discharge may not fully reflect long-term arrhythmic risk or eventual ICD eligibility (13, 15).

The wearable cardioverter defibrillator [WCD; LifeVest® (Zoll Medical Corporation, Pittsburgh, PA, USA)] was developed to provide temporary protection against SCD during this time-limited high-risk phase (16). The device continuously monitors cardiac rhythm and can deliver defibrillation therapy for sustained ventricular arrhythmias, offering a non-invasive option to bridge patients through the early recovery period before ICD eligibility is established (17). Evidence supporting WCD use includes the VEST randomised controlled trial (18) and subsequent as-treated analyses (19), as well as multiple European registries reporting high levels of adherence and favourable clinical outcomes in routine practice (20–22). The clinical benefit of WCD therapy appears closely linked to adherence, and real-world registry evidence suggests that high daily wear times are achievable in routine practice when appropriate patient education and follow-up are implemented (19, 20, 23, 24).

Several economic evaluations of WCD therapy have been published, demonstrating that cost-effectiveness is sensitive to baseline event risk, patient adherence, downstream ICD use, and healthcare system–specific costs (25–27). However, no analyses have been conducted in Spain, and the transferability of international economic evidence is limited by differences in clinical practice patterns, resource use, and decision-making frameworks (28).

The objective of this study was therefore to assess the cost-utility of the WCD in combination with GDMT compared with GDMT alone in post-MI patients with reduced LVEF at increased risk of SCD, from the perspective of the Spanish healthcare system. The cost-utility analysis was designed to reflect the Spanish clinical pathway for post-MI management, incorporating effectiveness estimates from the VEST as-treated analysis to represent outcomes among adherent patients (19), supplemented with real-world evidence to align model assumptions with routine clinical practice.

## 2 Methods

### 2.1 Study design

A cost-utility analysis was conducted to evaluate the use of a WCD in addition to GDMT, compared with GDMT alone, in patients at increased risk of SCD following MI. The analysis was based on a state-transition economic model representing the clinical course of patients during the early post-MI period and over the remainder of their lifetime. The model was developed in Microsoft Excel, Version 16.0, Microsoft Corporation, Redmond, WA, USA.

The methodological approach followed international best practice for economic evaluations, including the Consolidated Health Economic Evaluation Reporting Standards (CHEERS 2022) (29), and was aligned with national recommendations for economic evaluation issued by Spanish health authorities (30).

The model framework was originally developed for a previous economic evaluation conducted in another European healthcare system (26) and has been adapted for the present analysis by incorporating Spanish-specific cost data and, where available, clinical inputs relevant to Spanish clinical practice. Health outcomes were expressed in life-years gained and quality-adjusted life years (QALYs), and results were reported as incremental cost per QALY gained. Future costs and health outcomes were discounted at an annual rate of 3%, consistent with national recommendations (31).

### 2.2 Model structure and clinical pathway

The analysis used a Markov state-transition model with monthly cycles. A hypothetical cohort of 1,000 patients who had recently experienced an acute MI and had an LVEF of ≤35% entered the model at the time of hospital discharge.

At model entry, patients received either GDMT alone or GDMT plus a WCD during the initial post-MI period. During this early phase, patients were at risk of SCD, non-sudden death, and emergency implantation of an ICD following sustained ventricular arrhythmia.

After three months, corresponding to the medical optimisation period recommended in clinical guidelines (8), surviving patients were reassessed to determine eligibility for ICD implantation based on LVEF criteria. Patients meeting criteria transitioned to an ICD implantation state, while those not meeting criteria continued long-term management without device implantation.

**Figure 1:**
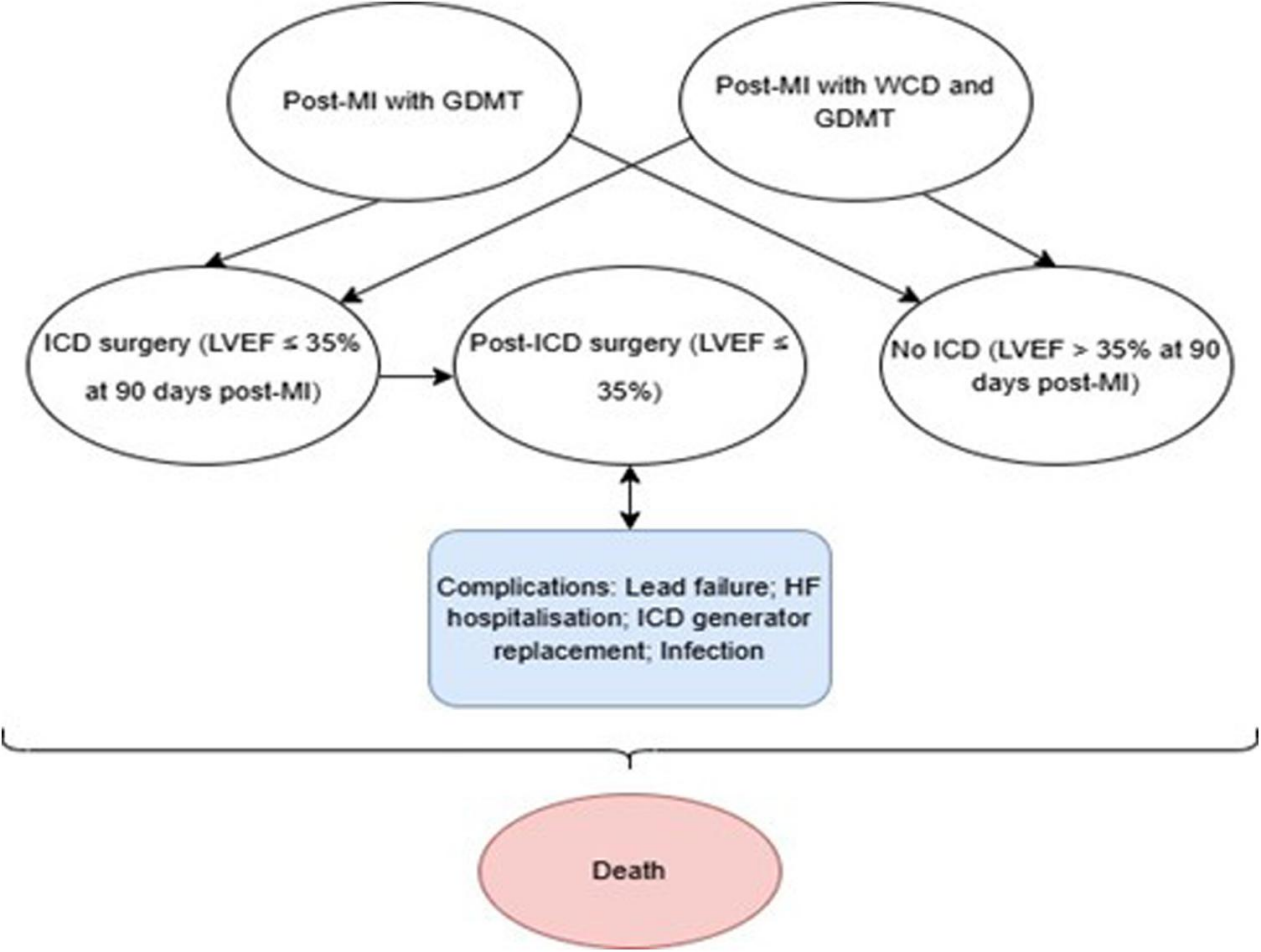
Markov model structure. GDMT, guideline-directed medical therapy; HF, heart failure; ICD, implantable cardioverter defibrillator; LVEF, left ventricular ejection fraction; MI, myocardial infarction; WCD, wearable cardioverter defibrillator.

Following ICD implantation, patients entered a post-implantation state in which they were at risk of device-related complications, including lead failure, infection and generator replacement, as well as hospitalisation for HF related to the underlying ventricular dysfunction. After resolution of these events, patients returned to the post-ICD state. Patients who received a WCD or an ICD experienced a probability of an inappropriate shock, which could result in hospitalisation. Death from cardiac or non-cardiac causes could occur from any health state and was modelled as an absorbing state. The model structure is illustrated in Figure 1.

### 2.3 Clinical effectiveness and transition probabilities

Clinical effectiveness during the early post-MI period was informed primarily by the as-treated analysis of the VEST randomised trial (19), which estimated mortality outcomes using observed WCD use as a time-dependent covariate. This approach was selected because the clinical benefit of the WCD is dependent on actual wear time, and adherence-adjusted estimates were considered more informative for modelling the effectiveness of WCD therapy under routine clinical use than intention-to-treat (ITT) estimates alone. However, given the observational nature of these estimates, uncertainty was explored through scenario analyses using per-protocol-based effectiveness estimates (19). These data were used to parameterise short-term survival in patients receiving WCD plus GDMT and those receiving GDMT alone.

Transition probabilities for emergency ICD implantation during the early post-MI period, recovery of ventricular function, and long-term mortality were derived from published observational studies and clinical trials reflecting contemporary practice (18, 32–39). Because Spanish data suitable for parameterising these transition probabilities were limited, the model used international published sources for key clinical inputs, while applying a clinical pathway consistent with contemporary guideline-based post-MI management in Spain. Specifically, probabilities of ICD implantation during the first 3 months after MI were informed by Dreher et al. (32), and the proportions of patients with LVEF ≤ 35% at 90 days were informed by Duncker et al. (40) for the WCD arm and Sjöblom et al. (41) for the GDMT-alone arm.

Long-term cardiac mortality was modelled separately for patients with and without ICD implantation, while non-cardiac mortality was based on age- and sex-specific Spanish population life tables (42). Risks associated with ICD implantation and device-related complications were also incorporated. A detailed summary of clinical input parameters and data sources is provided in Supplementary Table S1.

### 2.4 Healthcare costs and resource Use

The analysis was conducted from the perspective of the Spanish public healthcare system and included only direct medical costs. Cost categories included WCD acquisition and monitoring during the initial post-MI period, acute and chronic post-MI management, ICD implantation and replacement, management of device-related complications, hospitalisations, long-term care for patients with neurological impairment, and end-of-life care.

Cost inputs were obtained from Spanish national sources and published literature where available and were expressed in 2025 euros. When Spanish data were unavailable, values were informed by international sources and adapted to the Spanish context. A detailed presentation of unit costs and resource use assumptions is provided in Supplementary Table S2.

### 2.5 Health-related quality of life

Health outcomes were expressed as QALYs using utility values derived from published data. Baseline utilities for a Spanish cohort (43) were adjusted for age and sex and modified by disutilities associated with MI, ICD implantation, device-related complications, inappropriate shocks, and neurological impairment. Utility inputs and associated disutility values are presented in Supplementary Table S3.

### 2.6 Base-case, sensitivity, and scenario analyses

Base-case results were calculated using mean parameter values. Deterministic sensitivity analyses (DSA) were performed to assess the impact of uncertainty in key clinical and economic inputs by varying parameters across their respective 95% confidence intervals, where available. Probabilistic sensitivity analysis (PSA) was conducted using Monte Carlo simulation (10,000 model iterations), with appropriate probability distributions assigned to model parameters. Results were presented using cost-effectiveness scatter plots and cost-effectiveness acceptability curves.

In addition, a scenario analysis was conducted using a more conservative estimate of short-term mortality based on the per-protocol population of the VEST trial (19). In this scenario, 90-day all-cause mortality risks were derived from the randomised treatment groups reported in the trial (1.2% in the WCD plus GDMT group and 4.7% in the GDMT alone group). These cumulative probabilities were converted to monthly transition probabilities, resulting in monthly mortality probabilities of 0.0040 for the WCD plus GDMT arm and 0.0159 for the GDMT alone arm. This scenario was included to explore the impact of using a more conservative effectiveness estimate that does not adjust for adherence.

## 3 Results

### 3.1 Base-case analysis

Over a lifetime horizon, the use of WCD plus GDMT was associated with greater effectiveness and higher total costs compared with GDMT alone (Table 1).

**Table 1:** Results of base-case cost-effectiveness analysis over lifetime horizon.

**Costs and effectiveness**	**WCD + GDMT**	**GDMT alone**	**Incremental**
Life Years			
Optimisation Phase	0.28	0.26	0.02
Post-optimisation phase	12.55	12.04	0.51
**Total**	**12** **.** **83**	**12** **.** **30**	**0** **.** **53**
**QALYs**			
Optimisation Phase	0.24	0.22	0.02
Post-optimisation phase	9.35	8.94	0.41
**Total**	**9** **.** **59**	**9** **.** **16**	**0** **.** **43**
**Costs**			
Medical treatment	€16,434	€6,052	€10,382
ICD-related cost	€42,305	€42,121	€185
ICD-related complications	€13,231	€13,213	€18
non-ICD-related complications	€15,648	€15,504	€143
Follow-up costs	€12,218	€11,695	€522
End of Life	€7,471	€7,700	-€229
**Total**	**€113,290**	**€102,032**	**€11,259**
ICER/QALY	€26,145	-	-
Net monetary benefit^a^	€1,660	-	-

GDMT, guideline-directed medical therapy; ICER, incremental cost-effectiveness ratio; QALY, quality-adjusted life year; WCD, wearable cardioverter defibrillator.

a Using €30,000 WTP threshold.

Bold values indicate the total accumulated life years, QALYs, and costs for each treatment arm over the model time horizon.

In the base-case analysis, WCD plus GDMT generated a total of 12.83 life years compared with 12.30 life years with GDMT alone, corresponding to an incremental gain of 0.53 life years. After adjustment for health-related quality of life, WCD plus GDMT resulted in 9.59 QALYs compared with 9.16 QALYs with GDMT alone, yielding an incremental gain of 0.43 QALYs. Gains in both life years and QALYs were observed during the initial medical optimisation phase and were maintained over the post-optimisation period.

Total lifetime costs were higher in the WCD plus GDMT strategy (€113,290 per patient) than with GDMT alone (€102,032 per patient), resulting in incremental costs of €11,259. Higher costs in the WCD group were primarily driven by medical treatment costs during the early post-MI period and follow-up costs, while differences in ICD-related costs and complication costs were small between strategies. End-of-life costs were lower in patients receiving WCD plus GDMT.

Based on these results, the incremental cost-effectiveness ratio (ICER) for WCD plus GDMT compared with GDMT alone was €26,145 per QALY gained. This value lies below the commonly accepted willingness-to-pay (WTP) threshold of €30,000 per QALY (31), suggesting that WCD plus GDMT may be considered cost-effective under the assumptions of the model. At a WTP threshold of €30,000 per QALY, the corresponding net monetary benefit was €1,660.

### 3.2 Sensitivity and scenario analyses

**Figure 2:**
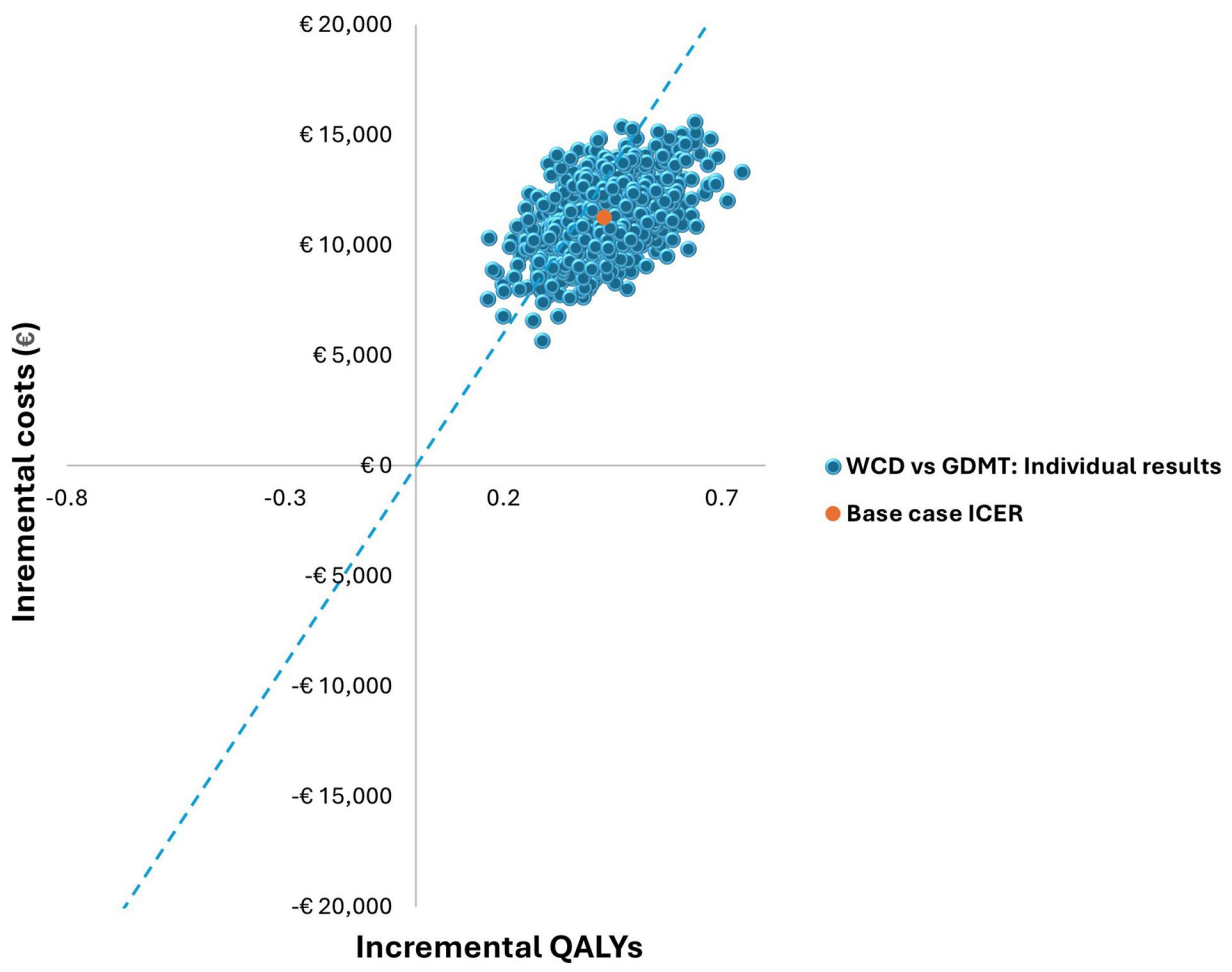
Cost-effectiveness plane. GDMT, guideline-directed medical therapy; ICER, incremental cost-effectiveness ratio; QALY, quality-adjusted life year; WCD, wearable cardioverter defibrillator.

**Figure 3:**
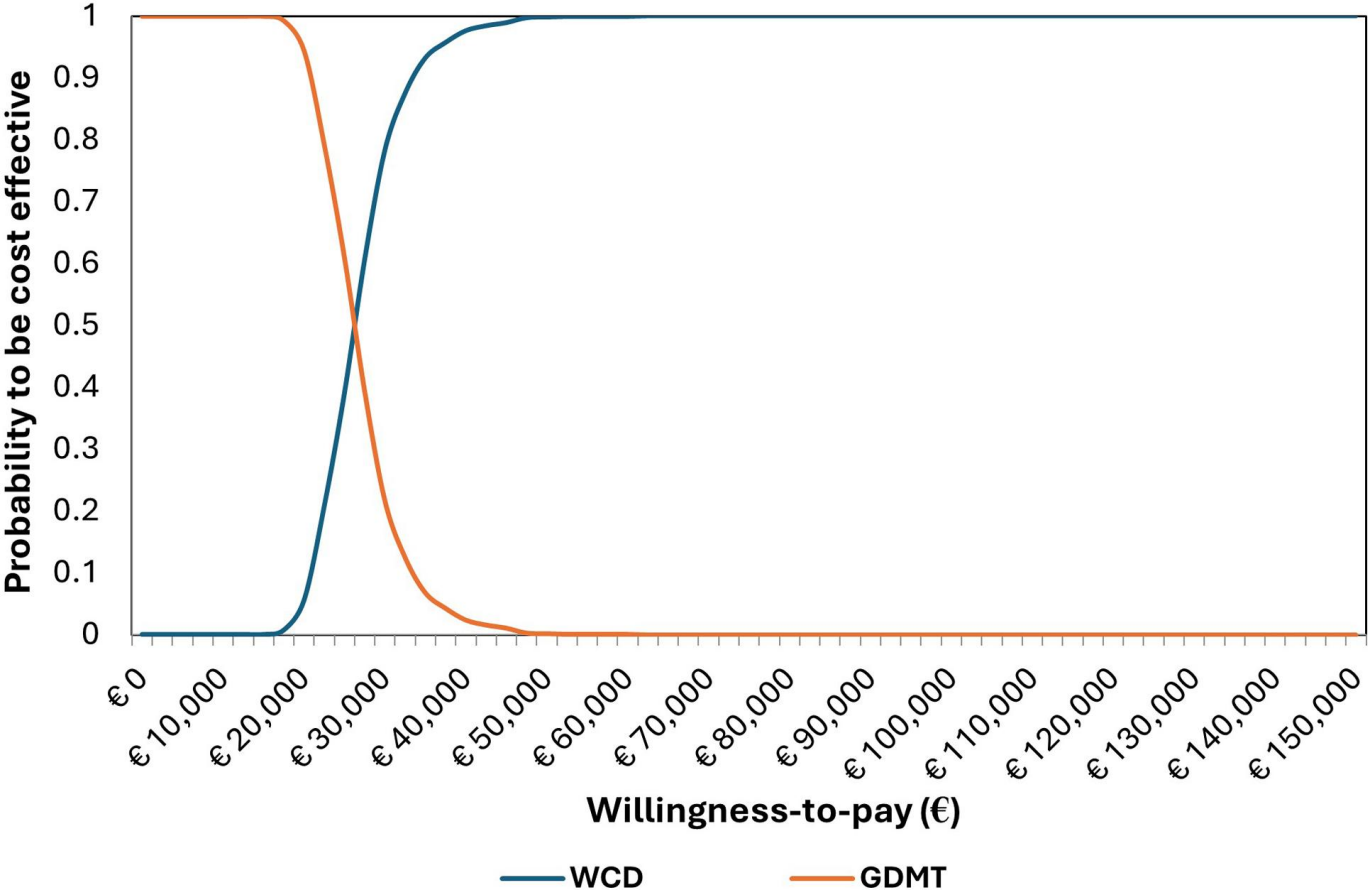
Cost-effectiveness acceptability curve. GDMT, guideline-directed medical therapy; WCD, wearable cardioverter defibrillator.

Results of the PSA are presented using the cost-effectiveness plane (Figure 2) and the cost-effectiveness acceptability curve (Figure 3). Across 10,000 simulations, most iterations were located in the north-east quadrant of the cost-effectiveness plane, indicating higher costs and greater effectiveness for WCD plus GDMT compared with GDMT alone.

In the probabilistic analysis, WCD plus GDMT was associated with mean discounted costs of €113,659 and 9.62 QALYs, compared with €102,437 and 9.19 QALYs for GDMT alone, resulting in an incremental cost of €11,222 and an incremental gain of 0.43 QALYs. The corresponding probabilistic ICER was €26,043 per QALY gained. At a WTP threshold of €30,000 per QALY gained, the probability of WCD plus GDMT being cost-effective was 78.3%.

**Figure 4:**
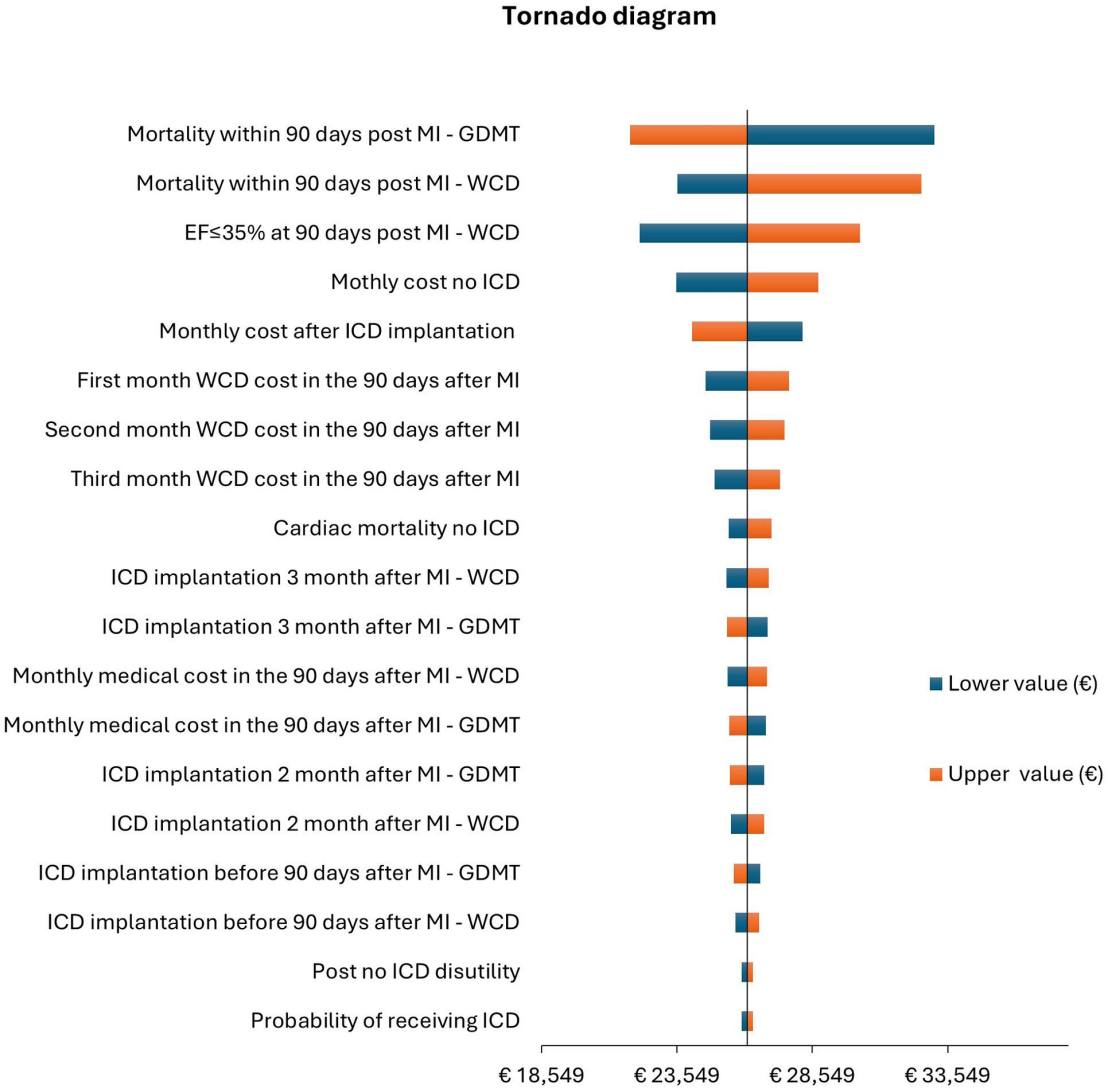
Deterministic sensitivity analysis (tornado diagram). EF, ejection fraction; GDMT, guideline-directed medical therapy; ICD, implantable cardioverter defibrillator; MI, myocardial infarction; WCD, wearable cardioverter defibrillator.

Results of the DSA are summarised in the tornado diagram (Figure 4). The analysis showed that the ICER was most sensitive to assumptions related to short-term mortality during the first 90 days after MI in both treatment arms. In particular, variation in mortality within 90 days post-MI for patients receiving GDMT alone and for those receiving WCD plus GDMT had the greatest impact on the ICER.

Other influential parameters included the proportion of patients with LVEF ≤ 35% at three months post-MI in the WCD plus GDMT arm and assumptions related to monthly costs in patients not receiving an ICD and following ICD implantation. In contrast, variation in WCD acquisition costs, probabilities of ICD implantation at different time points, utility values, and long-term cardiac mortality had a relatively smaller impact on the ICER. Across the vast majority of one-way sensitivity analyses, the ICER remained below commonly cited WTP thresholds (31), indicating that the base-case results were generally robust within the ranges explored in one-way sensitivity analyses.

In a scenario analysis using a more conservative estimate of short-term mortality derived from the per-protocol population of the VEST trial (19), WCD plus GDMT resulted in an incremental gain of 0.37 QALYs and an incremental cost of €10,707, yielding an ICER of €29,038 per QALY gained (Table 2). This ICER remained below the commonly cited WTP threshold of €30,000 per QALY.

**Table 2:** Results of scenario analysis over lifetime horizon.

**Costs and effectiveness**	**WCD + GDMT**	**GDMT alone**	**Incremental**
Life Years			
Optimisation Phase	0.28	0.26	0.02
Post-optimisation phase	12.59	12.17	0.43
**Total**	**12** **.** **87**	**12** **.** **43**	**0** **.** **45**
QALYs			
Optimisation Phase	0.24	0.22	0.02
Post-optimisation phase	9.38	9.03	0.35
**Total**	**9** **.** **62**	**9** **.** **25**	**0** **.** **37**
Costs			
Medical treatment	€16,474	€6,137	€10,337
ICD-related cost	€42,433	€42,504	-€71
ICD-related complications	€13,271	€13,332	-€61
non-ICD-related complications	€15,695	€15,646	€49
Follow-up costs	€12,256	€11,808	€448
End of Life	€7,453	€7,645	-€193
**Total**	**€113,585**	**€102,878**	**€10,707**
ICER/QALY	€29,038	–	–
Net monetary benefit^a^	€355	–	–

GDMT, guideline-directed medical therapy; ICER, incremental cost-effectiveness ratio; QALY, quality-adjusted life year; WCD, wearable cardioverter defibrillator.

a Using €30,000 WTP threshold.

Bold values indicate the total accumulated life years, QALYs, and costs for each treatment arm over the model time horizon.

In probabilistic analysis under this scenario, the probability of WCD plus GDMT being cost-effective at a WTP threshold of €30,000 per QALY was 57.2%. These findings indicate that, while the cost-effectiveness results are sensitive to assumptions regarding short-term mortality during the early post-MI period, WCD plus GDMT remains within the range of cost-effectiveness under more conservative assumptions.

## 4 Discussion

This study provides Spanish-specific economic evidence on the value of temporary protection with a WCD in patients with reduced LVEF following MI. When adapted to Spanish clinical practice patterns and unit costs, the analysis suggests that WCD use in addition to GDMT is associated within the model with projected gains in life years and QALYs at an incremental cost that falls below commonly cited WTP thresholds (31). The high probability of cost-effectiveness observed in PSA supports these findings within the assumptions of the base-case model.

A scenario analysis using more conservative effectiveness estimates derived from the per-protocol population of the VEST trial (19) resulted in a modestly higher ICER and a lower probability of cost-effectiveness, although the ICER remained below the commonly cited WTP threshold, reinforcing the sensitivity of the results to assumptions regarding early mortality.

Beyond cost-effectiveness alone, the broader clinical utility of the WCD has been highlighted in studies demonstrating its role in preventing arrhythmic deaths during the early post-MI period when patients are ineligible for ICD implantation, supporting the clinical rationale for its inclusion in acute risk management pathways (18, 20). Additionally, registry data indicate that early identification of ventricular arrhythmias — a key driver of WCD benefit — correlates with rapid initiation of further therapy, which may contribute to improved downstream outcomes (23, 24).

From a clinical perspective, the WCD occupies a clearly defined position within the contemporary post-MI care pathway, providing temporary protection during a period when patients remain at elevated arrhythmic risk but are not yet candidates for permanent device implantation according to guideline recommendations, or may ultimately not require ICD implantation if LVEF improves significantly following GDMT (8, 44). The modelled benefits were partly driven by reductions in early mortality, consistent with evidence that the highest risk of SCD occurs in the weeks to months following MI before ventricular function has stabilised (14, 15). DSA and scenario analysis indicated that assumptions related to short-term mortality during this early phase were the dominant drivers of cost-effectiveness.

Real-world implementation of WCD therapy is commonly supported by structured patient training and follow-up, which appear important for achieving high daily wear times in routine practice. European and international registry studies, including WEARIT-France, WEARIT-II, and SCD-PROTECT, have reported high adherence and daily wear times exceeding 22 h in many cohorts (22, 23, 40). Preliminary Spanish experience has also suggested comparable adherence, with a mean daily wear time of 22.8 h reported in the first 200 patients treated nationally (45). Together, these findings support the assumption that clinical benefit is closely linked to adherence and provide important context for interpreting effectiveness estimates derived from adherence-adjusted analyses in the model.

While adherence is an important determinant of effectiveness, patient selection also plays a key role in clinical decision-making. The eligibility criteria used in the model are broadly consistent with those of the VEST trial, which enrolled post-MI patients with reduced LVEF during the early recovery period (18, 19). However, treatment decisions in routine clinical practice can be more nuanced in variable circumstances. In clinical practice, both eligibility and expected benefit are likely to be influenced by additional factors, including arrhythmic burden, clinical stability, comorbidities, extent of myocardial scar, biomarker profile, and response to early medical therapy. As emerging evidence evolves there is an opportunity for future models to incorporate multiparametric risk stratification as the evidence base matures (46).

In economic terms, the present findings are consistent with cost-utility analyses conducted in other healthcare systems, while highlighting the importance of national context. Previous evaluations from Italy, England, and Poland have shown that WCD cost-effectiveness is sensitive to baseline event risk, ICD implantation patterns, and healthcare system–specific costs (25–27). By incorporating Spanish unit costs and reflecting local care pathways, this study addresses the limited transferability of international economic evidence and provides estimates that are more directly applicable to decision-making within Spain.

It is important to recognise that differences in hospitalisation costs, outpatient care patterns, and device pricing—all of which vary nationally—can significantly influence model outputs. For example, studies in high-income European settings have shown that reduced device acquisition costs and higher arrhythmic event rates lead to more favourable ICERs, highlighting how differences in key inputs shape economic conclusions (26). This contextual nuance supports the need for country-specific modelling rather than reliance on extrapolated international data.

The decentralised organisation of the Spanish healthcare system further strengthens the relevance of locally grounded analyses. In Spain, healthcare decision-making responsibilities are distributed across autonomous communities, and this structure can influence the timing and adoption of new cardiovascular technologies, reinforcing the value of country-specific economic evidence based on Spanish pathways and cost structures (47). Regional health technology assessment bodies increasingly require evidence that reflects Spanish clinical practice and resource use to inform funding and adoption decisions (48). In this context, the results of the present study may be particularly informative for regional evaluations, including those focused on optimising post-MI care pathways and reducing preventable SCD.

Short-term effectiveness estimates for WCD use were derived from the as-treated analysis of the VEST trial (19), which was considered appropriate because WCD benefit depends on actual device wear time (49). This analysis accounted for wear-time as a time-dependent variable and adjusted for predictors of adherence (notably diabetes and prior PCI), reducing—but not eliminating—the potential for bias. As with any adherence-adjusted analysis, residual confounding or selection effects may persist because patients who consistently wear the device could differ systematically from less-adherent individuals. Accordingly, the modelled treatment effect may represent the upper bound of benefit relative to an ITT estimate, and a moderate level of uncertainty remains when interpreting adherence-adjusted effects. However, real-world evidence suggests that adherence in routine clinical practice is often higher than that observed in VEST, where median wear time was approximately 18 h per day (18), with more recent international studies reporting wear times exceeding 22–23 h per day (22, 40, 45, 50, 51). This suggests that some events in the VEST intervention arm may have occurred during periods without device protection, and that adherence-adjusted estimates may better reflect the effectiveness of WCD therapy as implemented in current practice. This uncertainty was further explored in a scenario analysis using effectiveness estimates derived from the per-protocol population of the VEST trial (19), which resulted in a modestly higher ICER and lower probability of cost-effectiveness compared with the base-case analysis, while remaining within commonly cited WTP thresholds.

Some parameters were informed by observational data and non-Spanish sources because of limited availability of local long-term data. In particular, probabilities relating to ICD implantation and persistence of LVEF ≤ 35% at 90 days were derived from international studies (32, 40, 41) because equivalent Spanish data suitable for model parameterisation were not identified. These uncertainties were explored through sensitivity analyses, and the overall conclusions remained stable across a wide range of plausible assumptions.

Additionally, while the model incorporates a comprehensive set of clinical events, it does not explicitly capture broader patient-reported outcomes associated with WCD use, such as reassurance, anxiety, or treatment burden during the early post-MI period. These factors may influence health-related quality of life but are rarely quantified in economic evaluations of temporary device-based prevention strategies. Future research incorporating patient-reported outcomes could help clarify the broader impact of WCD therapy beyond survival and major clinical events.

Importantly, the model does not assume that WCD use directly improves ventricular function. Rather, it reflects the clinical benefit of preventing fatal arrhythmic events during a vulnerable period while allowing time for optimisation of medical therapy and appropriate reassessment of long-term device eligibility. In this respect, the findings support the WCD as a complementary, time-limited strategy within established post-MI management pathways. Beyond the prevention of SCD, it is also worth acknowledging the potential additional benefits associated with WCD use. Structured patient education and close remote monitoring during the wearing period may contribute to improved patient engagement and adherence to medical therapy. Moreover, some studies have reported improvements in quality of life measures in this population, which may represent an added value not fully captured by the model (52).

The framing of WCD as a bridge therapy aligns with guideline narratives emphasising the temporality of arrhythmic risk immediately following MI and supports its role in a comprehensive risk mitigation strategy that includes optimisation of GDMT and lifestyle interventions (8, 44). Moreover, as newer pharmacological agents and approaches to ventricular remodelling continue to evolve, the role of interim protective strategies such as WCDs remain relevant, especially in subgroups with persistently elevated arrhythmic risk despite optimal therapy.

## 5 Conclusions

In this Spanish cost-utility analysis, temporary use of a WCD in addition to GDMT was associated with projected improvements in survival and quality-adjusted life expectancy compared with medical therapy alone. When evaluated using Spanish-specific cost inputs, the resulting ICERs were below commonly cited WTP thresholds in both the base-case and scenario analyses.

These findings are sensitive to model assumptions, particularly those relating to early mortality, although WCD use remained within accepted cost-effectiveness thresholds under more conservative assumptions.

These results support consideration of WCD therapy within the post-MI care pathway in Spain, particularly for patients at transiently high risk of SCD and likely to achieve adequate adherence. Further evidence from real-world implementation and refined patient selection strategies may help to better define its value in routine clinical practice.

## Data Availability

The original contributions presented in the study are included in the article/Supplementary Material, further inquiries can be directed to the corresponding author.
