# Supplementary material for: Cost-utility analysis of the wearable cardioverter defibrillator in high-risk post-myocardial infarction patients in the Spanish healthcare system
Source: Front Cardiovasc Med. 2026 May 29;13:1832880. doi: 10.3389/fcvm.2026.1832880 (PMC13259661; doi:10.3389/fcvm.2026.1832880)
Supplement: Supplementary file 1 [file Datasheet1.pdf]

Supplementary Materials

Table S1 Clinical Input Parameters

Parameter	Value	Distribution	Source
**Population**			
Mean age	60.93	-	Olgin et al. (1)
Male patients (%)	73.00	-	Olgin et al. (1)
**Clinical efficacy**			
Medical optimisation phase (Post-MI with WCD)			
Mortality up to 90 days post-MI (monthly)	0.0050	Beta (10.3, 2,044.2)	Olgin et al. (1)
ICD implantation 1 month after MI	0.1100	Beta (88.9, 719.2)	Dreher et al. (2)
ICD implantation 2 months after MI	0.1100	Beta (88.9, 719.2)	Dreher et al. (2)
ICD implantation 3 months after MI	0.1100	Beta (88.9, 719.2)	Dreher et al. (2)
EF ≤ 35% at 90 days post-MI	0.3800	Beta (62.0, 101.2)	Duncker et al. (3)
Inappropriate shock within 90 days post-MI	0.0020	Beta (99.8, 50,199.1)	Olgin et al. (4)
Cardiac arrest within 90 days post-MI	0.0087	Beta (99.1, 11,294.1)	Olgin et al. (4)
Medical optimisation phase (Post-MI without WCD)			
Mortality up to 90 days post-MI (monthly)	0.0191	Beta (65.0, 3,336.4)	Olgin et al. (1)

ICD implantation 1 month after MI	0.1430	Beta (85.6, 512.7)	Dreher et al. (2)
ICD implantation 2 months after MI	0.1430	Beta (85.6, 512.7)	Dreher et al. (2)
ICD implantation 3 months after MI	0.1430	Beta (85.6, 512.7)	Dreher et al. (2)
EF ≤ 35% at 90 days post-MI	0.4070	Beta (59.3, 86.4)	Sjöblom et al. (5)
Cardiac arrest within 90 days post-MI	0.0087	Beta (99.1, 11,294.1)	Olgin et al. (4)
Post-medical optimisation phase – ICD surgery (EF ≤ 35% after 90 days post-MI)			
Death from cardiac arrest	0.0019	Beta (99.8, 52,480.8)	Greenberg et al. (6)
ICD procedural death	0.0020	Beta (99.8, 50,199.1)	Van Rees et al. (7)
Death from lead failure	0.0129	Beta (98.7, 7,552.21)	Cheng et al. (8)
Death from ICD infection	0.0500	Beta (94.9, 1,804.1)	Margey et al. (9), Sohail et al. (10)
Death from HF hospitalisation	0.0124	Beta (98.7, 7,864.8)	Shahar et al. (11)
Lead failure	0.0015	Beta (99.8, 66,465.8)	Woo et al. (12)
Lead infection	0.0007	Beta (99.9, 142,656.2)	Woo et al. (12)
Battery life (replacement)	0.0017	Beta (98.8, 59,800.2)	Olsen et al. (13)
Hospitalisation for HF	0.0080	Beta (99.2, 12,245.8)	Moss et al. (14), Tang et al. (15)
ICD inappropriate shock	0.0149	Beta (98.5, 6,490.9)	Turakhia et al. (16)
Cardiac arrest	0.0042	Beta (99.6, 23,378.9)	Bardy et al. (17)
Post-medical optimisation phase – No ICD surgery (EF > 35% after 90 days post-MI)			

Death from cardiac arrest	0.0026	Beta (99.7, 37,955.8)	Hall et al. (18)
Probability of receiving an ICD	0.0093	Beta (99.1, 10,559.6)	Goldstein et al. (19)
Cardiac arrest	0.0026	Beta (99.7, 38,260.8)	Hall et al. (18)

Abbreviations: EF, ejection fraction; HF, heart failure; ICD, implantable cardioverter defibrillator; MI, myocardial infarction.

Table S2 Healthcare Costs and Resource Use Parameters

Parameter	Value	Distribution	Source
**Costs**			
First month WCD cost	€3,400	Gamma (-343,300, 343,199)	ZOLL Medical España, S.L (20)
Second month WCD cost	€3,400	Gamma (-343,300, 343,199)	ZOLL Medical España, S.L (20)
Third month WCD cost	€3,400	Gamma (-343,300, 343,199)	ZOLL Medical España, S.L (20)
Monthly medical cost in the 90 days after MI - WCD	€475.17	Gamma (-47,892 47,791)	McDonagh et al. (21), McDonagh et al. (22)
Monthly medical cost in the 90 days after MI - GDMT	€475.17	Gamma (-47,892 47,791)	McDonagh et al. (21), McDonagh et al. (22)
Cost of ICD implantation	€18,547.16	Gamma (-1,873,163, 1,873,062)	Ministerio de Sanidad, Consumo y Bienestar Social 2025 (23)
Cost of ICD replacement	€18,547.16	Gamma (-1,873,163, 1,873,062)	Ministerio de Sanidad, Consumo y Bienestar Social 2025 (23)
Cost of battery replacement	€7,703.31	Gamma (-777,934, 777,833)	Ministerio de Sanidad, Consumo y Bienestar Social 2025 (24)
Mean cost per patient due to lead problem	€6,654.26	Gamma (-671,980, 671,879)	Ministerio de Sanidad, Consumo y Bienestar Social 2025 (25)

Cost due to infection-related ICD surgery	€12,709.80	Gamma (-1,283,590, 1,283,489)	Ministerio de Sanidad, Consumo y Bienestar Social 2025 (26)
Monthly cost after ICD implantation	€206.00	Gamma (-20,706, 20,605)	Servicio de Evaluación y Planificación del Servicio Canario de la Salud (SESCS) 2018 (27), Agència de Qualitat i Avaluació Sanitàries de Catalunya (AQuAS) 2020 (28)
Monthly cost patients EF > 35% no ICD	€206.00	Gamma (-20,706, 20,605)	Servicio de Evaluación y Planificación del Servicio Canario de la Salud (SESCS) 2018 (27), Agència de Qualitat i Avaluació Sanitàries de Catalunya (AQuAS) 2020 (28)
Monthly long-term care costs for patients with neurological impairment	€5,009.94	Gamma (-505,904, 505,803)	Gates et al. (29)
End-of-life costs	€12,995.00	Gamma (-1,312,395, 1,312,294)	Delgado et al. (30)

Hospitalisation due to cardiac arrest	€7,428.49	Gamma (-750,177, 750,077)	Ministerio de Sanidad, Consumo y Bienestar Social 2025 (31)
Hospitalisation due to inappropriate shock	€4,044.42	Gamma (-408,386, 408,285)	Ministerio de Sanidad, Consumo y Bienestar Social 2025 (32)
Hospitalisation due to HF	€4,044.42	Gamma (-408,386, 408,285)	Ministerio de Sanidad, Consumo y Bienestar Social 2025 (32)
Intensive Care Unit visit	€1,984.90	Gamma (-200,375, 200274)	Consejería de Salud y Consumo (Junta de Andalucía) 2024 (33)
GP visits (both in a surgery and at home)	€53.51	Gamma (-5,305, 5,205)	Consejería de Salud y Consumo (Junta de Andalucía) 2024 (33)
Cardiologist visit	€137.74	Gamma (-13,812, 13,711)	Consejería de Salud y Consumo (Junta de Andalucía) 2024 (33)
Electrophysiologist visit	€137.74	Gamma (-13,812, 13,711)	Consejería de Salud y Consumo (Junta de Andalucía) 2024 (33) (Assumed same as cardiologist visit)
Tests (including blood tests, urea, creatinine and electrolytes)*	€40.99	Gamma (-4,040, 3,941)	Consejería de Hacienda, Presupuestos y Asuntos

			Europeos (Gobierno de Canarias) 2023 (34), Institut Català de la Salut 2024 (35)

Abbreviations: EF, ejection fraction; GDMT, guideline-directed medical therapy; GP, general practitioner; HF, heart failure; ICD, implantable cardioverter defibrillator; MI, myocardial infarction; WCD, wearable cardioverter defibrillator.

Table S3 Health-Related Quality of Life Parameters

Parameter	Value	Distribution	Source
Utility values			
Disutility of MI	0.0626	Beta (93.7, 1,402.8)	Sullivan et al. (36) Applied for 91 days
Disutility of ICD surgery	0.0493	Beta (95.0, 1,833.1)	Smith et al. (37) Applied for 3 days
Disutility post-ICD (EF ≤ 35%)	0.1167	Beta (88.2, 667.7)	Sullivan. (36) Applied long-term
Disutility post-no ICD (EF > 35%)	0.0368	Beta (96.3, 2,520.1)	Sullivan. (36) Applied long-term
Disutility of lead failure	0.0657	Beta (93.4, 1,327.5)	Feingold et al. (38) Applied for 2 days
Disutility of infection	0.1643	Beta (83.4, 424.3)	Feingold et al. (38) Applied for 5 days
Disutility of ICD replacement	0.0493	Beta (95.0, 1,833.1)	Smith et al. (37) Applied for 3 days
Disutility of HF hospitalisation	0.1904	Beta (80.8, 343.4)	McMurray et al. (39) Applied for 12 days
Disutility of inappropriate shock	0.0082	Beta (99.2, 11,974.8)	Sanders. (40) Applied for 0.5 days
Disutility of SCA	0.1643	Beta (83.4, 424.3)	Smith et al. (37) Applied for 10 days

Disutility of long-term care post-SCA	0.6100	Beta (38.4, 24.5)	Javanbakht et al. (41), Gage et al. (42), Raina et al. (43) Applied long-term

Abbreviations: EF, ejection fraction; HF, heart failure; ICD, implantable cardioverter defibrillator; MI, myocardial infarction; SCA, sudden cardiac arrest.

References

1. Olgin JE, Lee BK, Vittinghoff E, Morin DP, Zweibel S, Rashba E, et al. Impact of wearable cardioverter-defibrillator compliance on outcomes in the VEST trial: As-treated and per-protocol analyses. J Cardiovasc Electrophysiol. 2020 May 3;31(5):1009–18. doi:10.1111/jce.14404

2. DREHER TC, EL-BATTRAWY I, RÖGER S, ROSENKAIMER SL, GERHARDS S, KUSCHYK J, et al. Comparison of the Outcome of Patients Protected by the Wearable Cardioverter Defibrillator (WCD) for <90 Wear Days *versus* ≥90 Wear Days. In Vivo (Brooklyn). 2020 Nov 3;34(6):3601–10. doi:10.21873/invivo.12205

3. Duncker D, König T, Hohmann S, Bauersachs J, Veltmann C. Avoiding Untimely Implantable Cardioverter/Defibrillator Implantation by Intensified Heart Failure Therapy Optimization Supported by the Wearable Cardioverter/Defibrillator—The PROLONG Study. J Am Heart Assoc. 2017 Jan 11;6(1). doi:10.1161/JAHA.116.004512

4. Olgin JE, Pletcher MJ, Vittinghoff E, Wranicz J, Malik R, Morin DP, et al. Wearable Cardioverter–Defibrillator after Myocardial Infarction. New England Journal of Medicine. 2018 Sep 27;379(13):1205–15. doi:10.1056/NEJMoa1800781

5. Sjöblom J, Muhrbeck J, Witt N, Alam M, Frykman-Kull V. Evolution of Left Ventricular Ejection Fraction After Acute Myocardial Infarction. Circulation. 2014 Aug 26;130(9):743–8. doi:10.1161/CIRCULATIONAHA.114.009924

6. Greenberg H, Case RB, Moss AJ, Brown MW, Carroll ER, Andrews ML. Analysis of mortality events in the multicenter automatic defibrillator implantation trial (MADIT-II). J Am Coll Cardiol. 2004 Apr;43(8):1459–65. doi:10.1016/j.jacc.2003.11.038

7. van Rees JB, de Bie MK, Thijssen J, Borleffs CJW, Schalij MJ, van Erven L. Implantation-Related Complications of Implantable Cardioverter-Defibrillators and Cardiac Resynchronization Therapy Devices. J Am Coll Cardiol. 2011 Aug;58(10):995–1000. doi:10.1016/j.jacc.2011.06.007

8. Cheng A, Wang Y, Curtis JP, Varosy PD. Acute Lead Dislodgements and In-Hospital Mortality in Patients Enrolled in the National Cardiovascular Data Registry Implantable Cardioverter Defibrillator Registry. J Am Coll Cardiol. 2010 Nov;56(20):1651–6. doi:10.1016/j.jacc.2010.06.037

9. Margey R, McCann H, Blake G, Keelan E, Galvin J, Lynch M, et al. Contemporary management of and outcomes from cardiac device related infections. Europace. 2010 Jan 1;12(1):64–70. doi:10.1093/europace/eup362

10. Sohail MR, Uslan DZ, Khan AH, Friedman PA, Hayes DL, Wilson WR, et al. Management and Outcome of Permanent Pacemaker and Implantable Cardioverter-Defibrillator Infections. J Am Coll Cardiol. 2007 May;49(18):1851–9. doi:10.1016/j.jacc.2007.01.072

11. Shahar E, Lee S, Kim J, Duval S, Barber C, Luepker R V. Hospitalized heart failure: rates and long-term mortality. J Card Fail. 2004 Oct;10(5):374–9. doi:10.1016/j.cardfail.2004.02.003

12. Woo CY, Strandberg EJ, Schmiegelow MD, Pitt AL, Hlatky MA, Owens DK, et al. Cost-Effectiveness of Adding Cardiac Resynchronization Therapy to an Implantable Cardioverter-Defibrillator Among Patients With Mild Heart Failure. Ann Intern Med. 2015 Sep 15;163(6):417–26. doi:10.7326/M14-1804

13. Olsen T, Jørgensen OD, Nielsen JC, Thøgersen AM, Philbert BT, Johansen JB. Incidence of device-related infection in 97 750 patients: clinical data from the complete Danish device-cohort (1982–2018). Eur Heart J. 2019 Jun 14;40(23):1862–9. doi:10.1093/eurheartj/ehz316

14. Moss AJ, Hall WJ, Cannom DS, Klein H, Brown MW, Daubert JP, et al. Cardiac-Resynchronization Therapy for the Prevention of Heart-Failure Events. New England Journal of Medicine. 2009 Oct;361(14):1329–38. doi:10.1056/NEJMoa0906431

15. Tang ASL, Wells GA, Talajic M, Arnold MO, Sheldon R, Connolly S, et al. Cardiac-Resynchronization Therapy for Mild-to-Moderate Heart Failure. New England Journal of Medicine. 2010 Dec 16;363(25):2385–95. doi:10.1056/NEJMoa1009540

16. Turakhia MP, Zweibel S, Swain AL, Mollenkopf SA, Reynolds MR. Healthcare Utilization and Expenditures Associated With Appropriate and Inappropriate Implantable Defibrillator Shocks. Circ Cardiovasc Qual Outcomes. 2017 Feb;10(2). doi:10.1161/CIRCOUTCOMES.115.002210

17. Bardy GH, Lee KL, Mark DB, Poole JE, Packer DL, Boineau R, et al. Amiodarone or an Implantable Cardioverter–Defibrillator for Congestive Heart Failure. New England Journal of Medicine. 2005 Jan 20;352(3):225–37. doi:10.1056/NEJMoa043399

18. Hall TS, von Lueder TG, Zannad F, Rossignol P, Duarte K, Chouihed T, et al. Relationship between left ventricular ejection fraction and mortality after myocardial infarction complicated by heart failure or left ventricular dysfunction. Int J Cardiol. 2018 Dec;272:260–6. doi:10.1016/j.ijcard.2018.07.137

19. Goldstein SA, Li S, Lu D, Matsouaka RA, Rymer J, Fonarow GC, et al. Implantable Cardioverter Defibrillator Utilization and Mortality Among Patients ≥65 Years of Age With a Low Ejection Fraction After Coronary Revascularization. Am J Cardiol. 2021 Jan;138:26–32. doi:10.1016/j.amjcard.2020.09.056

20. ZOLL Medical España SL. Suministro de alquiler de chalecos desfibriladores cardioversores portátiles para el servicio de Cardiología del Hospital Universitario de Bellvitge (Expediente No. CS/AH02/1101425381/25/PNSP, Anexo 18: Oferta Final) [PDF] [Internet]. 2025 [cited 2026

Feb 6]. Available from: https://contractaciopublica.cat/portal-api/descarrega-document/301341335/5129298655031A647433FD08B6DD062A

21. McDonagh TA, Metra M, Adamo M, Gardner RS, Baumbach A, Böhm M, et al. Guía ESC 2021 sobre el diagnóstico y tratamiento de la insuficiencia cardiaca aguda y crónica. Rev Esp Cardiol. 2022 Jun;75(6):523.e1-523.e114. doi:10.1016/j.recesp.2021.11.027

22. McDonagh TA, Metra M, Adamo M, Gardner RS, Baumbach A, Böhm M, et al. 2023 Focused Update of the 2021 ESC Guidelines for the diagnosis and treatment of acute and chronic heart failure. Eur Heart J. 2023 Oct 1;44(37):3627–39. doi:10.1093/eurheartj/ehad195

23. Ministerio de Sanidad C y BS. Portal Estadístico del SNS – Ministerio de Sanidad, Consumo y Bienestar Social (DRG 179) [Internet]. 2025 [cited 2026 Feb 6]. Registro de altas. Grupos Relacionados por el Diagnóstico (GRD-APR) – CMBD 2023. Available from: https://pestadistico.inteligenciadegestion.sanidad.gob.es/publicoSNS/S

24. Ministerio de Sanidad C y BS. Portal Estadístico del SNS – Ministerio de Sanidad, Consumo y Bienestar Social (DRG 176) [Internet]. 2025 [cited 2026 Feb 6]. Registro de altas. Grupos Relacionados por el Diagnóstico (GRD-APR) – CMBD 2023. Available from: https://pestadistico.inteligenciadegestion.sanidad.gob.es/publicoSNS/S

25. Ministerio de Sanidad C y BS. Portal Estadístico del SNS – Ministerio de Sanidad, Consumo y Bienestar Social (DRG 177) [Internet]. 2025 [cited 2026 Feb 6]. Registro de altas. Grupos Relacionados por el Diagnóstico (GRD-APR) – CMBD 2023. Available from: https://pestadistico.inteligenciadegestion.sanidad.gob.es/publicoSNS/S

26. Ministerio de Sanidad C y BS. Portal Estadístico del SNS – Ministerio de Sanidad, Consumo y Bienestar Social (DRG 711) [Internet]. 2025 [cited 2026 Feb 6]. Registro de altas. Grupos Relacionados por el Diagnóstico (GRD-APR) – CMBD 2023. Available from: https://pestadistico.inteligenciadegestion.sanidad.gob.es/publicoSNS/S

27. Servicio de Evaluación y Planificación del Servicio Canario de la Salud (SESCS). Informe de evaluación del Servicio de Evaluación del Servicio Canario de la Salud (SESCS) sobre el DAI subcutáneo (2018): Evaluación de efectividad clínica y coste del DAI subcutáneo frente al DAI transvenoso. 2018.

28. Agència de Qualitat i Avaluació Sanitàries de Catalunya (AQuAS). Desfibrilador automático implantable para prevención primaria de la muerte súbita cardíaca en España: eficacia, seguridad y eficiencia [Internet]. 2020 [cited 2026 Feb 6]. Available from: https://scientiasalut.gencat.cat/bitstream/handle/11351/5794/desfibrilador_automatico_implantable_prevencion_primaria_muerte_subita_cardiaca_espana_eficacia_seguridad_eficiencia_2020.pdf

29. Gates S, Lall R, Quinn T, Deakin CD, Cooke MW, Horton J, et al. Prehospital randomised assessment of a mechanical compression device in out-of-hospital cardiac arrest (PARAMEDIC): a pragmatic, cluster randomised trial and economic evaluation. Health Technol Assess (Rockv). 2017 Mar;21(11):1–176. doi:10.3310/hta21110

30. Delgado JF, Oliva J, Llano M, Pascual-Figal D, Grillo JJ, Comín-Colet J, et al. Health Care and Nonhealth Care Costs in the Treatment of Patients With Symptomatic Chronic Heart Failure in Spain. Revista Española de Cardiología (English Edition). 2014 Aug;67(8):643–50. doi:10.1016/j.rec.2013.12.014

31. Ministerio de Sanidad C y BS. Portal Estadístico del SNS – Ministerio de Sanidad, Consumo y Bienestar Social (DRG 196) [Internet]. 2025 [cited 2026 Feb 6]. Registro de altas. Grupos Relacionados por el Diagnóstico (GRD-APR) – CMBD 2023. Available from: https://pestadistico.inteligenciadegestion.sanidad.gob.es/publicoSNS/S

32. Ministerio de Sanidad C y BS. Portal Estadístico del SNS – Ministerio de Sanidad, Consumo y Bienestar Social (DRG 194) [Internet]. 2025 [cited 2026 Feb 6]. Registro de altas. Grupos

Relacionados por el Diagnóstico (GRD-APR) – CMBD 2023. Available from: https://pestadistico.inteligenciadegestion.sanidad.gob.es/publicoSNS/S

33. Consejería de Salud y Consumo (Junta de Andalucía). Orden de 24 de mayo de 2024 - Boletín Oficial de la Junta de Andalucía (BOJA) (108). 2024 Jun.

34. Consejería de Hacienda P y AE (Gobierno de C. Resolución de 3 de enero de 2023 - Boletín Oficial de Canarias (BOC) (7). 2023 Jan.

35. Institut Català de la Salut. Resolució SLT/4583/2024, de 18 de desembre - Resolució SLT/4583/2024, de 18 de desembre - Diari Oficial de la Generalitat de Catalunya (DOGC) (9,315). 2024 Dec.

36. Sullivan PW, Slejko JF, Sculpher MJ, Ghushchyan V. Catalogue of EQ-5D Scores for the United Kingdom. Medical Decision Making. 2011 Nov 21;31(6):800–4. doi:10.1177/0272989X11401031

37. Smith T, Jordaens L, Theuns DAMJ, van Dessel PF, Wilde AA, Hunink MGM. The cost-effectiveness of primary prophylactic implantable defibrillator therapy in patients with ischaemic or non-ischaemic heart disease: a European analysis. Eur Heart J. 2013 Jan 1;34(3):211–9. doi:10.1093/eurheartj/ehs090

38. Feingold B, Arora G, Webber SA, Smith KJ. Cost-Effectiveness of Implantable Cardioverter-Defibrillators in Children With Dilated Cardiomyopathy. J Card Fail. 2010 Sep;16(9):734–41. doi:10.1016/j.cardfail.2010.04.009

39. McMurray JJ V, Trueman D, Hancock E, Cowie MR, Briggs A, Taylor M, et al. Cost-effectiveness of sacubitril/valsartan in the treatment of heart failure with reduced ejection fraction. Heart. 2018 Jun;104(12):1006–13. doi:10.1136/heartjnl-2016-310661

40. Sanders G, Owens D, Hlatky M. Potential Cost-effectiveness of Wearable Cardioverter-Defibrillator Early Post Myocardial Infarction. Journal of Innovation in Cardiac Rhythm Management. 2015;1929–40.

41. Javanbakht M, Mashayekhi A, Hemami MR, Branagan-Harris M, Keeble TR, Yaghoubi M. Cost-Effectiveness Analysis of Intravascular Targeted Temperature Management after Cardiac Arrest in England. Pharmacoecon Open. 2022 Jul 3;6(4):549–62. doi:10.1007/s41669-022-00333-7

42. Gage BF, Cardinalli AB, Owens DK. The effect of stroke and stroke prophylaxis with aspirin or warfarin on quality of life. Arch Intern Med. 1996 Sep 9;156(16):1829–36. PubMed PMID: 8790077.

43. Raina KetkiD, Callaway Clifton, Rittenberger JonC, Holm MargoB. Neurological and functional status following cardiac arrest: Method and tool utility. Resuscitation. 2008 Nov;79(2):249–56. doi:10.1016/j.resuscitation.2008.06.005
